# Dr. Ideler on Religious Insanity

**Published:** 1848-04-01

**Authors:** 


ART. III.—*Der religiöse Wahnsinn; erlautert durch Krankengeschichten. Ein Beitrag zur Geschichte der religiösen Wirren der Gegenwart.* Von Dr. KARL W. IDELER, Professor der Medizin und Lehrer der Psychiatrischen Klinik au der Friedrich-Wilhelms-Universität, &c. &c. Halle, 1847. 8vo, pp. 230.

*Religious Insanity, illustrated by Histories of Cases; a contribution to the History of the Religious Errors of the Age.* By Dr. K. W. IDELER, Professor of Medicine and Clinical Psychiatry at the University of Berlin, &c.

THE momentous results that depend upon a correct religious life and conversation, the transcendent importance of thinking and acting rightly as to religious doctrine, duties, and discipline, and the general demand for an answer to the oft-repeated question, "WHAT IS TRUTH?" give both a popular and scientific interest to all those various forms of insanity which involve religion and morals. The desire for eternal life is the fundamental characteristic of humanity; for although it may be considered as only a modification of the instinct of self-preservation and the sentiment of love of life arising therefrom, yet the desire itself is so wrapped up with a religious and spiritual consciousness, and has such profound and intimate relations to an unseen world of thought and morals, that its instinctive origin is lost entirely in its results.

The manifestations of this desire of a return to life after death are so numerous, that even a bare catalogue would hardly be contained in the space allotted to us. Its relations to the Creator, as the Lord and Giver of Life, are manifested in the multitudinous forms of religion that from time to time have appeared upon earth, in a form, it is true, according with the pre-existing habits of thought of the people, or the circumstances modifying them, but always pointing more or less distinctly to a spiritual governor of the universe, and an omnipotent, unseen Being, who can alike save or destroy as He wills. To such a centre of religious doctrine and feeling all inquirers have referred.

> "Father of all—in every age,
> In every clime adored;
> By saint, by savage, or by sage,
> Jehovah, Jove, or Lord."

The *natural* religion of man has never, however, been distinguished for grandeur or truth, and there is great force in the declaration that man makes his God like to himself. Let the man be bloodthirsty, cruelty is an attribute of his divinity; let the man be crafty or libidinous, such is his deity; let the man be profoundly philosophical, such is his God; and the attributes of the human finite mind are imputed to the infinite.

The object and aim of revealed religion is to modify the earthly and false principles which natural religion includes and promulgates; and the whole of what is termed religious education ought to be conducted with the view of bending the deductions of the untutored mind to the truths of revelation, and by a diligent circulation of sound doctrines eradicate the false; with how little success this has been done the pages of history amply prove. Fanaticism, folly, knavery, insanity, are traceable in the professors of every form of religious belief; very traceable in the false creeds, but too lamentably manifest in the true.

The history of the church of Christ, the great teacher and promulgator of His doctrines, and the means by which many successive generations of mankind have been taught religious truths, displays an immeasurable delineation of the most dangerous errors as the necessary results of a degenerate piety and an ignorant devotion; mad bigotry and fanaticism may be seen hand-in-hand, and darkening the fair doctrines and morals of Christian truth. The most frightfully destructive wars, the inconceivable cruelties, caused and practised by inexorable power, the deepest sagacity, and the maddest insanity, have been the means used on too many occasions to root out heresy, to promulgate dogmas, or to defend a degenerate and an erroneous faith. If we were to look only at the dark side of the picture which the history of Christianity presents, we might be readily tempted to pronounce it a curse rather than a blessing, and despair of human happiness.

To the psychologist, this dismal view of our holy religion becomes the more dismal when he finds, in scrutinizing the details of history, that often the heretic was only a religious madman, and that purgation by hellebore would have been the remedy for his heterodoxy rather than purification by fire. Knowing the all-engrossing nature of religion, and the intensity of the emotions and feelings that it excites, he is prepared, *à priori*, to expect every form of insane aberration from religious truth, and every form of mysticism and fanaticism. It enters every action, it tinges every thought, it is manifest in every deed, and while it is often the more intensely felt in the nervously susceptible, when intensely felt by the strong, it does not fail to leave its traces on the organism. How often religious excitement is mere animal excitement; how often religious insanity is excited by religion, and how often by functional or structural disease of the cerebrum, are important questions to solve, inasmuch as the solution involves not only the discrimination of what is religious truth, but also the determination of the ætiology and treatment of insanity in matters of religious belief and conduct.

Professor Ideler holds a prominent position in the medical school at Berlin, as a teacher of the theory and practice of that department of medicine which comprises mental disorders. His work consists of an Introduction of twenty-six pages, and the histories of nineteen cases of religious insanity occupying the remainder of the volume. In the Introduction, Professor Ideler developes a theory as to the nature of religious insanity which presents all the peculiarities of German modes of thought, and has therefore that transcendental and unpractical character which renders German theories distasteful to the more practical English mind. In connexion with this theory, and indeed as a principal object, he developes his ideas as to the nature of those observations of the human mind in matters of religion, which becoming manifest from time to time in great numbers of people, give them the characteristics of an epidemical disease, closely analogous to insanity, if not identical with it. And we apprehend that medical science may be of great service to religion and morals, by showing in this way this analogy or identity of fanaticism and mental aberration; many good well-meaning persons might be saved from themselves and their cerebral infirmities, and religion rendered the more holy and more useful by being freed from the stains and incumbrances with which every new development of it, in successive generations, is defiled and hindered by ignorant prejudices, and the fantastic dreaming and visions of the partially, if not wholly insane.

The histories of the cases recorded by Professor Ideler are well adapted to this purpose. They are elaborate and complete, and satisfactorily trace the morbid state of mind from the earliest outbreak of religious feeling to its progressive and complete development—from mental health to complete insanity. And it is only by such histories that the inquiring mind can be fully satisfied as to those simpler forms of insanity, in which the deviation from a healthy mental state appears rather like an earnest and a truthful enthusiasm than that which it really is. We purpose, therefore, to give such a general sketch of one or two of these cases as will exhibit their more peculiar points, and enable the reader to trace the progressive changes in the mind of the patients. We shall at the same time show the predisposing and exciting causes, and by so much, contribute to a more perfect prophylaxis and sounder therapeutics.

The first case is that of a patient, born in 1813, the son of a carpenter. From his infancy he was affected with scrofula, and, up to the age of sixteen, had chronic ophthalmia. He was consequently feeble and afflicted, never engaging in childish sports, but in manner grave and melancholy. The father was a drunkard, and abused the children: he had frequent quarrels with the mother in consequence, and the angry feelings thus excited at last brought on epilepsy, which first deprived her of reason, and finally of life, after sixteen years' duration.

In consequence of the long-continued ophthalmia, the patient went little to school, and scarcely acquired the elements of secular knowledge; but the religious teaching made a deep impression on his mind, so that he viewed the mimicry and mockery of the minister by other boys with lively displeasure, and often prayed earnestly to God to deliver his mother from her afflicting malady. He became timid and shy, and had a daily hæmorrhage from the nose from the age of sixteen to twenty-five, often accompanied by violent palpitation, sometimes alternating with headach, and sometimes with depressing emotions. He often felt so weak, that he feared lest he should fall in the street; or when he saw the announcements of deaths, he was seized with fear and trembling for his approaching end. He became so downcast, that he not only avoided all opportunities of amusement, but resigned himself to the conviction that God had appointed him to suffer, and he often burst into tears when the sun broke forth, at the thought that he was not worthy that the sun should shine upon him. In his eighteenth year he was apprenticed to be a carpenter; and whenever he had to walk upon rafters, he was seized with giddiness, and was obliged to creep along them on his hands and knees. The sudden death of a second master, to whom he was transferred, made a great and serious impression on his mind, and led him to think that he might also die suddenly in an unprepared state of mind, and his soul perish everlastingly.

When twenty-two, he went to Berlin, and got on well in business. He had now instead of the epistaxis a frequently recurring determination of blood to the head and chest, violent palpitation, intense headach, vertigo, and flashings before the eyes; and neither bleedings from the arm nor other means entirely relieved him. His mental troubles were still further increased by an indulgence in illicit pleasure; this was followed by bitter remorse, and in consequence, he was induced often to receive the sacrament. He had spectral illusions when seven years old, of a peculiar character, and these continued through life; at first, when he went to bed in the dark, he saw visions of human forms, the bodies of which were well-shaped, but the faces like those of spectres, and distorted in every possible way. For the most part the nose was long, the mouth wide open, the eyes large; there were commonly from four to six such figures, which were partly motionless, mostly of unknown persons, but occasionally of known, and disappeared in about five minutes. At first these phantoms had no expression, but subsequently they grinned, rolled their eyes, and made horrible grimaces. During later years they appeared during the day as well, particularly during the heat of summer, or when lifting a heavy weight, or when fear excited flashings before the eyes, vertigo, and palpitation. As he had experienced these spectral illusions from early infancy they attracted little of his attention, except when a sudden sensation of fear was excited by some peculiarly horrible grimaces, or when some dreadful ghost-story excited his imagination. He considered them usually as but phantoms of the imagination, and it was only with increasing religious terrors that he began to think them visions of the devil. In consequence of the long continuance of this morbid state, the spectres attained so great vividness, that he saw the figures of deceased people, as, for example, that of his mother, although long since dead, with the greatest distinctness.

For a series of years he continued in tolerable health, except when his conscientiousness and fear of death excited gloomy feelings. Insignificant circumstances brought on the most painful scruples of conscience—as, for example, trifling expenses on festive occasions, when he considered he had thoughtlessly expended the money that ought to have been given to the poor, or to his needy father. He thought his bodily infirmities were a punishment for his crimes; he would often go to church to pray for the forgiveness of his sins, and he entertained all kinds of religious scruples, which were depressing and melancholy in their general character. He wished to have his brother become professedly religious, and got him persuaded to receive the sacrament; previously to his going, however, the patient wished to wash his brother's feet, in imitation of Christ, who washed his disciples' feet.

Encouraged by his success with his brother, he next attempted the conversion of his sister; and it became a favourite object with him to reunite the whole family, and to have them to receive the sacrament together. The sister, however, received this proposition, and his pious exhortations, with sulky silence; she was a married sister, and, in his judgment, a very undutiful wife. This sister gave him so many renewed occasions to reprove her conduct, that he became quite unhappy, and passed his nights without sleep.

The patient so often sent money to his needy father, that he was as often without cash himself, and was obliged to borrow from his sister. One morning when he asked a loan of 8 groschen, (10*d.*) it was granted him so reluctantly that he was highly offended, and left the room. The sister followed him, and presented him the money with such an ugly grimace that he was terrified. At the same moment he observed an old broom placed against the wall, which, if met in the morning, according to an old superstition, indicated bad luck, and might even call up the devil himself. The thought came like a flash of lightning that his sister was the devil, and that he had taken her form to give him the money and thereby bind him to his service. Just at this time a piece of glass wounded his foot through his boot, and this he considered to have happened through the agency of the devil, who was continually meeting him. He now took to continual prayer, or perusal of the Bible, for assistance and consolation. A passage in the 10th chapter of the Acts of the Apostles, in which Peter addresses Cornelius to the effect that his prayer was heard, &c., suddenly excited the idea that God might be induced to hear his supplications if he were to fast strictly, and he resolved accordingly. He therefore fasted from Sunday to the Monday week following. During this time he felt no hunger, and only a little thirst on Sunday week after the fast commenced. He occupied his time with a continual perusal of the Bible, and with closed doors, not able to work, and resisting all solicitations to take food. His spectral illusions were more vivid than ever; they consisted in ugly devilish faces, and the whole of his insane ideas (which are detailed at length by Professor Ideler) had an almost exclusive reference to the devil. After reading, however, the announcement by Christ of the destruction of Jerusalem, he was convinced that the world was coming to an end, and that Berlin would be destroyed by fire. Under this impression he set forth, with a piece of wood as an amulet, to bless the houses of his friends, and thereby preserve them from the impending destruction. He walked before the house fixed upon for this purpose, murmuring—"In the name of God the Father, the Son," &c. Arriving at the Charité Hospital, he said the Lord's Prayer, blessed it, and feeling thirsty, drank at the fountain near. He went also to bless the hospital for invalided soldiers, to churches, and various private houses and public institutions. The day following, he was admitted into the Charité, and it was found necessary to feed him with a tube, lest he should starve to death. On the next day, hearing that the various members of his family were reconciled to each other, he considered that his prayers had been answered, and that the necessity for fasting no longer existed. Contrary to the earnestly expressed wishes of his physician, his father now took him home, and as a necessary result, the patient soon fell into his previous state of diablerie, began to exorcise, pray, &c., and shortly again refused food, in pursuance of his resolution to fast and pray against the devil and his machinations. His nights were sleepless, and he was harassed with the most horrid spectral figures of grinning devilish faces, with wide mouths, blazing rolling eyes, cornute brows, and the other fantastic characteristics with which artists love to designate the devil, and of which specimens may be seen in Teniers' painting, "The Temptation of St. Anthony."

The patient was a second time brought into the Charité. He was now sunk into a gloomy, contemplative silence, and took no notice of the external world, to which he appeared altogether dead. It was necessary to feed him with the elastic tube for six days; the cold douche to the head was then used, and he at last began to become conscious of surrounding objects, to sleep calmly, and to take his food. In eight months he was restored to his friends in a tolerable state of bodily and mental health.

A summary of the case which we have taken from Ideler's work, and presented to our readers in an abridged form, but without any very material omission, may not be uninteresting, especially if accompanied with an analysis of the predisposing and exciting causes, and the symptoms and cure of the affection. Of the predisposing causes we find, first, an hereditary tendency to cerebral disease derived from both parents. The long-continued series of epileptic attacks to which the mother was subject previously to her death, and which, in effect, proved ultimately fatal, on the one side; and, on the other, the habitual desire for alcoholic stimulants, show this predisposition sufficiently, for all experienced persons know that habitual intoxication is, in many cases, only another form of insanity. Secondly, an actually existing morbid condition of the brain, of a permanent character, is shown by the persistence of spectral illusions from childhood, by vertigo, spectral flashes of light, headach, &c. That there was congestion of the vessels of the head and face is shown by the occurrence of epistaxis, and this was probably connected with cardiac disease, of the existence of which we have a slight indication in the attacks of palpitation to which the patient was subject. Disease of the heart is a frequent concomitant of insanity; it is five times more prevalent among an insane population than the general population.

The existence of active scrofula during childhood manifests a congenital delicacy and susceptibility of the organism; all impressions, therefore, would be more influential with the patient than with persons of ordinary health; and hence we find that the cerebral phenomena alluded to above were always aggravated either by emotional stimuli, or the application of causes acting with a depressing influence on the system in general, and the nervous system in particular.

The instinctive faculties appear to have been highly developed. The same fear of death and of suffering, and its religious manifestation—the fear of punishment by eternal death,—are both leading traits in the character of the patient. Add to this timidity, the instinct of submission to authority to escape the anticipated evils, on which the religious life was based, and we have a clue to the conduct of the individual, and of the peculiar form of insanity manifested. The chronic ophthalmia which afflicted the patient until his sixteenth year prevented his regular attendance at school, and thereby that training of the mental faculties, and that development of the intellectual powers, which would have modified, if not over-ruled, the predominant instincts. For want of these, the development of the religious element of the man's character took the lowest form of religious manifestation, and the Deity was regarded as an avenging being, to be propitiated by prayers and intercessions, and a scrupulous attention to ceremonials. It was a modification of demon-worship. It was not surprising, then, that the spectral illusions of the patient were of a corresponding character, and took the form of horrible demoniacal spectres; that gloom and despair characterized the morbid mental condition; and that an importunate devil was ever present in the sufferer's thoughts, and only to be exorcised by biblical and supplicatory exercises.

The patient's anxiety to withdraw his family from the evils which his own too highly developed instincts led him to fear for himself, was only an offshoot from those instincts; it bore the same relation to self-preservation as the love of offspring bears.

The immediate predisposing causes of the paroxysm of insanity do not appear from the history, and have evidently been overlooked. All we learn is, that at a certain period of life the religious sentiment became more and more manifested, and the whole current of ideas turned into its vortex, until, progressing from one morbid state to another, the cerebral irritability sank into inirritability; the mental activity ceased, and life itself was only saved by the forcible administration of food. That the immediate or proximate cause of the disease was cerebral irritability was manifest from the therapeutics; the cold douche alone roused the slumbering reason.

This history is imperfect in this, that it omits to describe or refer to that large class of excitantia malorum comprising disorders of the organs to which the peripheral nervous system is distributed. We have no account of gastric, hepatic, or intestinal irritation; no account of the state of the generative organs, bladder, or skin.

The lessons to be drawn, as to the therapeutics of religious insanity, from this case, are for the most part obvious enough; we will therefore only refer to the prophylaxis. Excluding the actual morbid condition of the brain which existed in the chronic form, the most important predisposing cause was the want of a general education. Where there is already a predisposition to cerebral disease in a religious family,—that is to say, a family in which the doctrines and discipline of religion have a marked influence on their actions and habits,—it is a fatal mistake to encourage the religious sentiment in early infancy or childhood, and thereby render the youth precociously religious. The irregularity of development of the mental faculties that will necessarily arise out of this exclusively religious training, will as necessarily lead to irregularity of life and conduct, and the proverb be verified in the individual, "A young saint, an old devil." The true check to a morbid and predominant action of the religious sentiment is to be found in the study of secular science. Dr. Cheyne observes,* "If the exercise of the religious sentiments be interrupted by too exclusive an attention to science, communion with God will lose its relish. Claudius Buchanan, while at Cambridge, wrote to a friend as follows: 'I find the great attention to study has made me exceedingly languid in my devotional duties; I do not feel that delight in reading the Bible, nor that pleasure in divine things which formerly animated me. On this account have many students in this university wholly abandoned the study of mathematics, for it seems they generally feel the same effects that I do.' "

This result of the close study of mathematics has been observed before. The husband of the celebrated Mrs. Montague was an example. His want of belief was a great sorrow to his wife. Dr. Beattie in vain conferred with the expiring mathematician on the truths of Christianity, and observed, "he set too much value on mathematics, and piqued himself too much on his knowledge of that science." Other illustrations might readily be adduced. It is obvious that any pursuit which requires an habitual application of the strict laws of evidence, and disciplines the mind to an accurate estimate of things, will be opposed to all ideas founded on belief only. But as belief, or faith, is an essential element of the human mind, and as without it there can be no religion whatever, the exact sciences may be both an antidote and a bane. The cultivation of them may beneficially repress an exuberance of religious sentiment, but it may also extinguish a minimum amount, and so lead to another, and, perhaps, more destructive form of insanity—a form characterized by an utter abandonment of all religious and moral principles whatever, and by utter depravity.

A remark worthy of point in the history of this case is, the suddenness with which the ideas that determined the line of insane conduct were excited. While reading of the religious exercises of Cornelius, the Roman centurion, the idea is suddenly formed to do likewise, and its

* Essays on Partial Derangement of the Mind, in supposed Connexion with Religion, p. 58.

action is permanent, or at least remains on the mind until another predominant idea is as suddenly developed. This is a general thing in insanity. The excitants of these sudden ideas are various. Sometimes it is the sight of a material object. A mother, seeing a knife or a razor glittering before her, is seized with the almost irresistible impulse to slay her husband or child; or if upon a height, to throw herself or another headlong; or if on the brink of a river or lake, to plunge in. Sometimes it is spectral illusions that determine the line of marked action, as in the history before us, where the sister was deemed to be and treated as the devil. Sometimes voices will syllable names, or propose a line of conduct. A medical practitioner, lately deceased, informed Dr. Cheyne that his father was one day sent for to Mr. Cowper, (we presume the poet,) then labouring under an attack of fanatical insanity supported by auricular delusions, whom he found with a pen-knife sticking in his side, with which, conceiving that he had received a mandate from Heaven to that effect, he had made an effort to kill himself.* Sometimes mandates of this kind have the effect of determining a particular line of religious duty. A friend of our own, influenced by the oratory of the late Rev. Mr. Irving, and falling at last into religious insanity, received a mandate in a dream to go forth into India and preach the Gospel of Christ. He forthwith took steps to obey, with no other symptom of insanity than this act of obedience. He gave up his business, sold his furniture, &c., and prepared for his departure; but before he could embark, the disease had sufficiently progressed to show its true character. He thought handkerchiefs came from his abdomen, having the power to work miracles; his nights he spent in wrestling in prayer with legions of devils, and his days in preaching, Bible in hand, to any ragged lads or ragged people he could gather round him in the streets or thoroughfares of London.

The history of almost all religious sects affords abundant examples of spectral illusions suddenly influencing individuals to a determinate line of conduct, and of such mere cerebral disease being mistaken for demoniacal agency, or the operation of the Holy Spirit of God. That the Deity communes with man is certain; and not the less certain because multitudes hear him not; but we think the time is come when an important question of this kind should be put on its proper footing, so that our holy and intellectual religion may not be sullied by words of man's

* Cowper, under the influence of his attacks of melancholy despondency, made several attempts to destroy himself. On one occasion, after suffering from a paroxysm of hypochondriasis, he resolved to throw himself off the Tower-stairs into the river. For this purpose, late one evening he called a hackney coach from the stand, and desired the driver to take him to the Tower-stairs. The coachman drove off towards the city. For nearly two hours he was engaged in driving about the streets without stopping. At the expiration of this time the coach stopped at Cowper's house. The driver, upon being expostulated with, observed, that he had been in the habit of going to the Tower frequently during the week, and he was ashamed to say that he had in vain attempted that evening to find the place. Cowper got out of the carriage and returned to the solitude of his own chamber, and offered up a prayer to the Divine Being for thus specially interposing in his behalf. It was on the following morning that he composed the beautiful hymn commencing—

" God moves in a mysterious way
His wonders to perform,
He plants his footsteps on the sea,
And rides upon the storm."

invention, or obscured by the wild chaotic action of a distempered brain.

It is very usual to attribute religious insanity to religion itself. In the preceding case, however, no such imputation can rest; nay, it seems the rather probable that the strong religious sentiment of the man guided him aright when he would otherwise have failed, and it was rather the cerebral disease that aggravated this, than this that induced the cerebral disease and its manifestation—namely, religious insanity. In the succeeding case, however, we find a sort of religious excitement developing insanity.

A master shoemaker, aged 45, twice happily married, had been resident in Berlin twenty-three years as journeyman and master. He was a happy and contented tradesman, and so little given to religious duties that it was rarely he went to church. In the year 1842, a member of the sect of Anabaptists left him some mission tracts and journals, and he was induced to attend their meetings. In a while he became more intimate with the members, till at last the numerous services, both on Sunday and week-day, tasked him beyond his mental powers, and he compared his head to an overfull stomach. He became indignant against the practical opposition to worldly pleasures shown by the sect, against the disputations held in the congregation as to the true exposition of the Bible, and particularly against the oft-repeated dogma that other Christians could not be holy, since, as they had not received the true baptism, they could not live according to the Gospel. He was so shocked by these things, that he the more diligently frequented the forbidden church, and this might have warded off the impending attack of insanity, if the mysticism so frequently characteristic of fanatical sects had not entered already deeply into his mind. Notwithstanding his original aversion to the Anabaptists, he felt himself irresistibly attracted towards them, lent a ready ear to their doctrines, while emissaries were sent to him to confirm him in his tendencies. They taught him that baptism of adults, the example of which was set by the baptism of Christ in the river Jordan, could alone render a man truly pious, and save him from damnation. He now became more than ever occupied with religious matters; he was timid and undecided in his business, and was harassed with religious scruples such as had never occurred before, so that his early life appeared blameable, and caused him bitter remorse. To remedy this unhappy condition he diligently read his Bible even while working at his trade, as enjoined by his brother sectarians. Continually harassed by doubts, and vainly wrestling for a solution of them, he at last became so miserable as not to be able to follow his business, and even during the night had no refreshing sleep, for he was disturbed from rest by horrible dreams of spectres and ugly hags. At last he believed he had discovered a solution of all this in the Bible, and that the grace of God and a new spiritual birth by baptism were necessary to put away his sins. He now joined the Anabaptists, and, with fourteen others, bewailed his sins with groans and sighs, and deep regrets that he had not served God from his youth, &c.

On April 29th, 1842, at six o'clock in the morning, he went with the other candidates for baptism to the Kummelsburg lake, about a quarter of a (German) mile from Berlin, on the shore of which two tents were erected for the two sexes to undress in. Every one had to strip to his shirt and put on a blouse over it with a girdle. Previously to the baptism, a prayer was offered up, a psalm sung, and a text read from the New Testament, to which the preacher appended an exhortation. This finished, each candidate was led into the lake, the preacher taking him by the girdle with his left hand, and with his right thrusting his head under water, while he said, "I baptize thee in the name," &c. The ceremony was not, however, calculated to have a beneficial effect, since it excited a variety of mingled emotions. The sobs and cries of the females (amongst whom was his own daughter, aged fifteen) disturbed his devotion, together with the feeling of shame in having to undress publicly and be exposed to a crowd of curious spectators. His teeth chattered with cold, and he was glad to get home with the happy consciousness that he was now born again.

This state of peace did not, however, last long; he soon got into doubts and difficulties, then quarrelled with his brethren, and becoming anabaptistically heterodox, was excommunicated. He then became convinced that the Anabaptists were judaizing Christians, and wrote to the precentor accordingly, and finally found his way back to the evangelical church.

After awhile he again got into controversy with his former co-religionists, and while under great consequent excitement, wrote an "Answer to the Encyclical Letter of Pope Gregory XVI. from Rome, May 23, 1844." This composition commenced with correctly expressed, although desultory remarks, but it soon became incoherent and unintelligible, and the reader vainly attempts to discover a meaning. We subjoin an example.

"Thou XVI. Cross-father, thou placed thy X on the Trinity, then remained to thee three 666, and the one man, Christ, thou crucified, who lived here 33 years—666; and since thou crucified the Trinity, who was the 33 years' man, (Revelations xiii., ver. 18,) one over the other, so it is—

| | | |
|---|---|---|
| God the Father, | Son, | and Holy Ghost, |
| 10, | 10, | 10, |
| Hay, | Straw, | Stubble. |

Mammala, will we go home? Let one have bite at the crumb of the loaf, then we go home. Rev. xi. ver. 4; xi. ver. 7. Conclusion Rev. xi. ver. 8. Yes, we have tormented you," &c.—P. 32.

After awhile he began to have dangerous notions. He wanted to force his wife through the window; he took a knife to slay his children, as Abraham did to Isaac. At last, on the evening of March 17, 1845, he became raving mad. He broke the furniture to pieces, tore up the beds, and, between whiles, danced, sang, drummed, and called after his children as they fled from his violence—"Quick, quick, a kiss." He tore his clothes off to his shirt, then wrapped a table-cloth round his body, and girded his chest so tightly with a towel that he could hardly breathe. We need not detail his other fantastic tricks; in a word, he became a raving maniac; and when the paroxysm passed away, he sank into listlessness.

After various means were tried unsuccessfully, tartar-emetic ointment was applied to the shaven scalp with the happiest results; and in a few months he was restored to his family.

In this case we have a series of phenomena in remarkable contrast with those of the preceding. The patient appears to have been a good and kind husband and father, but without any religious predisposition or turn of mind. It is only when the stimulus is accidentally applied by the proselyting tracts of the Anabaptists, that there is action excited. The enthusiastic character of the tracts would have an abiding influence in a mind untrained in religious discipline or to religious habits; and thus that morbid condition of the brain was excited, which, with a less stimulation and a more judicious communication of religious views, would have led only to a religious life and conversation.

The morbid condition took on the form of irritation of a chronic kind; and it appears as if it had spread downwards until it involved the instincts, the organs of which are situate laterally, and at the base of the brain. We thus find him running wildly, and destroying both inanimate things and animate. What the predisposing causes were do not appear; but there must, we think, have been something to assist the action of the Anabaptistical enthusiasm. That the latter was the *exciting* cause is obvious enough.

It were a most ungracious task to designate analogous examples to this as occurring amongst many protestant sects, and not unfrequently amongst Roman-catholic devotees. We feel, however, that it is incumbent upon us to notice the mental aberrations that have been excited by so-called popular preachers; for the results are equally injurious to religion and morals, as well as to the unfortunate subjects of the false enthusiasm. It appears to us that clergymen, ministers, and priests, should more diligently study the history of religious insanity, and so be enabled to discriminate accurately between the ravings of the insane, or semi-insane, and the operations of the Holy Spirit. It is, we think, a most dangerous error to confound the one with the other—dangerous to the eternal welfare of the teacher, who will not be held blameless at the great and awful day of the Lord, if he have neglected to inform his mind rightly on the subject; and dangerous to the poor deluded sufferer, whose mental life and spiritual prospects are thus prematurely blighted. Men consider too seldom that, as regards eternal life, and a preparation for it, the access of incurable insanity is virtually death; and, consequently, that the wild preachings of the enthusiast have as deadly an influence on his victim's future state, as if, by means of arsenic, he had been sent to his account "with all his imperfections on his head."

It will, perhaps, be thought well to analyze this question more minutely, and trace it to its elements. The question is of sufficient importance in this age of religious inquiry and excitement. On a careful perusal of those histories which modern literature affords of the great periods of religious excitement and enthusiasm, and of those minor fermentations which have led to the establishment of new sects, or have excited local manifestations of religious aberration, we find this general principle manifested in all—namely, that the minds of men have been directed to one dogma, or doctrine, or point of discipline in especial, either to the total eclipse of other doctrines, or to their partial obscuration and neglect. And for the most part, it may farther be stated, as an important fact, that the dogma, or principle, or point of discipline to which this prominent position is assigned, is generally of secondary importance in the general estimate of Christendom. For example, while all Christians maintain, with one exception, that baptism is an essential rite, one sect attributes to it a sacramental efficacy, while another maintains it is only a point of discipline or a ceremony, which may be varied according to circumstances; or, to refer to a circumstance in the last case, one sect may think pædobaptism is the important thing, while another may assert the absolute necessity to salvation of adult baptism. Now, the common faith of all these sects is, that, abstractedly, baptism is necessary to salvation by Christ; and this, therefore, is a fundamental principle of their Christian belief; yet none assert this fundamental principle. What distinguishes each is, that baptism shall be performed in a particular way, at a particular age, and with the belief in certain dogmas of less importance than the fundamental principle itself. We will mention an illustration or two of this point, as it is one of some importance in the prophylaxis and therapeutics of religious insanity, and cannot be too deeply impressed on the mind, or too clearly stated.

When the Reformation had nearly established itself in Germany, and men's minds had been agitated by religious strifes, about the year 1525, the whole country was disturbed by some turbulent preachers. The ringleader was one Thomas Muncer, "who, pretending a more than ordinary zeal, (having with much passion preached against the popish errors,) at length began to preach against Luther, terming him as too cold, and his sermons as not savouring enough of the Spirit. With great earnestness he pressed the exercises of mortification, and exhorted to a more frequent and familiar conversation with God; he pretended to some divine revelations, and that God, by dreams and visions, did reveal unto his saints his will." At length he became so mad, that he told his followers he had received a command from God to kill and root up all wicked princes and magistrates. He was a mischievous maniac, like the Kentish Thom. Muncer was the first of the Anabaptists who also held certain quaker tenets, as that it was not lawful for Christians to go to law, or to swear, &c.; and at first the members of the sect were orderly persons.

A tailor of Leyden went to live at Munster, and privately taught the doctrine of re-baptization, and won many converts amongst the lower orders. When an attempt was made to confute him and his co-religionists, several went running about the streets, crying, "Repent, and be re-baptized, lest the wrath of God overwhelm you." After a while they got worse in their insanity, and began to teach practices and doctrines very like those of the Mormonites of the present day. Prophets and prophetesses began to arise, and revelations from God were plentiful. John of Leyden, the tailor, (amongst others,) betaking himself to sleep, continued in a dream for three days together. Being awakened, "He speaks not a word, but calls for paper; in it he writes the names of twelve men, who were to be chief officers over God's Israel, and to govern all things; for such," he said, "was the will of the heavenly Father when he had thus prepared the way to his kingdom." He propounded, as a revelation from heaven, that no man was bound to one wife only, but that every man might have as many as he pleased. John took three; and those were considered to be the most pious and praiseworthy who had the greatest number. One of John's queens (for he was made king) thinking that it was not pleasing to God that men should die of famine, as they did during the siege of Munster, ventured to express her opinion, for which John led her into the market-place, and commanding her to kneel down, struck off her head.

The wildest doctrines were preached, and every part of Holy Writ applied to the most absurd ends by these insane fanatics; and the wars were made abundantly serviceable in palliating or defending every kind of cruelty and pillage. At last the ringleaders were taken and tortured to death; and their historian in the Harleian Miscellany concludes his history of their proceedings (how vainly history tells) with, "So let all the factious and seditious enemies of the church and state perish, but upon the head of King Charles let the crown flourish. Amen."

This account of the Anabaptists was entitled, "A Warning for England, especially for London," and was written in 1642, during the civil war. It is not a remarkable circumstance that the warning was unheeded, and that every kind of religious insanity (or fanaticism, which is only madness with a method) should so soon become rampant. Nay, in Cromwell there was a man of the same order of mind as John of Leyden, although more powerful and less deranged.

It is not always, however, that the influence of religious excitement upon one topic terminates in an insane excitement of the *destructive* propensities. We have seen that amongst the Anabaptists the *sexual* passion was also active; but this, in some sects, has been a leading trait. In the Harleian Miscellany (vol. iii.) there is an account of a sect of this kind termed "The Family of Love," printed in 1641, an age of fanaticism. It is doubtful whether the facts related be true—they are indecent enough—but the relater became insane, and the pamphlet is interesting as significant of the current ideas at the time. In all ages, indeed, this instinctive aberration has followed on religious insanity, and entered largely into the celebration of "mysteries."

The vast majority of modern sects do not, however, go so far as those of the stirring times above-mentioned. The religiously insane of these sects seek usually for some spiritual or temporal benefit to themselves by fasting, prayer, penance, &c., amongst one class; and by an earnest desire for conversion, or spiritual gifts and graces, amongst another. Occasionally the ceremonial most approved is a travestie of some point in Jewish history, as with the Shakers or Jumpers. In almost all examples of this form of religious insanity, it has appeared as an epidemic or endemic. In Hecker's Account of the Epidemics of the Middle Ages, there is also an interesting history of certain epidemics of religious insanity.

The way in which these epidemics arise is the same as that in which religious insanity occurs in individuals. Some general cause directs men's minds to religious thoughts and feelings—as a plague, a famine, an earthquake, or popular preachers, or religious dissensions and controversies. The excitement is in proportion to the force of the impulse, the amount of restraining power in the mass, and the general susceptibilities. The general excitement, although at first chaotic, soon separates into distinct forms or masses, or rather, the people affected form into groups around some idea common to all as a nucleus. In proportion as the constituents of these groups are people of low manners, uneducated, and vulgar, in that proportion will the morbid phenomena be rhapsodical and incoherent as to speech, and violent and brutish as to action. Superstition, cunning, avarice, lasciviousness, revenge will be predominant. In the more highly educated person there will be more of refined mysticism, more subtle superstition, and more delicacy in speech and manner. The larger majority will have brains sufficiently healthy to resist the effects of the excitement at this stage, and the irritation will not pass into congestion or inflammation. Those, however, who may be predisposed to cerebral disease, or with a highly susceptible nervous system, will be apt to become truly insane, the subjects of dreams, visions, and revelations; and, finally, exhausting the whole nervous force, a calm succeeds, and the disease ends in health or imbecility, as circumstances may arise. Now, the true preventive against all these evils rests in a great degree with the teachers of religion and the conductors of public worship. Instead of joining the insane fanaticism of the half-educated and the ignorant, they should manfully resist it by every proper means. They should avoid all controversial topics, all exalting services, all enthusiastic methods of teaching or preaching. The precepts of Christianity, rather than the doctrines, should be the theme of their discourses; and great moral duties rather than sectarian peculiarities. In calling the attention of members of the sacred profession to this important subject, we feel that we are only following in the footsteps of our common Teacher and Saviour. Jesus Christ pointed out very clearly how religious fanaticism and fanatical errors would arise in times of great political excitement. We would refer in particular to the thirteenth chapter of St. Mark's Gospel, for an illustration. When nation rises against nation and kingdom against kingdom, and earthquakes in divers places, and famines and troubles, and yet only "the beginnings of sorrows;"—in those days of intense affliction, Christ declares, false Christs and false prophets shall rise, and shall show signs and wonders. But he adds, "If any man shall say to you, Lo, here is Christ, or lo, he is there, believe him not. Take ye heed; behold, I have foretold you all things." Such is the emphatic warning of Him who spake as never man spake; and in his footsteps should his ministers and people tread.

It will hardly, we hope, be thought that we advocate the doctrines just stated as applicable to religious insanity exclusively. One idea is always a dangerous idea to the man of weak or inharmonious cerebral development, of whatever nature it may be, or to whatever applied. Indeed, it only arises after the premonitory stage of insanity has commenced, in a large proportion of cases. But the exclusive application of the mind will induce this premonitory or introductory stage—the stage of incubation. The enthusiastic patentee of a new discovery, the devoted lover, the absorbed mathematician, the devourer of novels, or books of travels, the overworked politician or litterateur—all these have afforded examples of the baneful influence exercised on the brain when the thoughts flow in one current through it, and the faculties of the mind are brought unequally into exercise. Mental gymnastics are as necessary as corporal, else the mind becomes diseased or distorted.

The third case in Professor Ideler's work will illustrate some of our remarks. A female, the daughter of a mechanic, who was in narrow circumstances, and sought comfort in religious exercise from the troubles of poverty, was early taught religious views, and to exercise herself in devotional services. Her mother died when she was young, and a step-mother rendered her life miserable by scolding and beating her; so that the world was as a waste to her, and she had great mental distress. Her father dying, she had to go into service; but her bad bodily health enabled her to do but little. Her bowels were obstinately confined, menstruation irregular, her eyes inflamed, her head ached, &c., and she only attained a tolerable state of health when menstruation was fully established, at the age of twenty-three. Being servant at an ale-house, the brutality of the guests so disgusted her, that she went to learn dress-making, and engaged, for the purpose of solace and support under her trials, in religious devotions, self-examination, and a strict asceticism. She was so industrious at her employment, and so attentive to her duties at a Sunday-school, that she had no time for amusement, and, in consequence, her health became indifferent. Eager after continual religious excitement, she seized every opportunity to induce and increase it, and was not only a constant attendant at church and pietistic meetings, but she had a number of pious sayings printed on her memory. Nevertheless, her hardly gained peace of mind was soon disturbed by the perusal of divers fanatical and mystical tracts. She spent her nights without sleep, and was anguished both night and day. She one evening saw visions in the sky of a religious cast, and after a sleepless night went to church, where she suddenly heard an inward voice say to her, "Thou art a Jew, and must be baptized." She was much alarmed, and knew not what to think; on her way home, she saw the Saviour as he is risen sitting on the right hand of God the Father, but his head was bent, and he seemed to suffer, so she remembered, that "without holiness no man shall see the Lord." She considered these visions to be from God for her comfort and the establishment of her faith.

Nothing more than the strictest attention to the duties of religion seems to have been observed for nine years after this. At this time, she was in the house of a Lutheran, who strenuously maintained his church to be that in which salvation was to be obtained, and that all persons not in it would be damned. A shoemaker who used to join their society launched the most violent anathemas against the Anabaptists, and said that they went about in the forms of angels, but the devil was behind them. The violent denunciations of those two primitive Lutherans had a powerful effect on her mind, and induced her to inquire more into the tenets and worship of the Anabaptists, and was so pleased with them that she attended their services. The question was now raised whether she should be re-baptized or not, and she devoted much time to prayers and perusal of the Bible, and long disputations with members of the sect. She had again spectral illusions, one of which was the full figure of an Anabaptist who had tried to persuade her to become one of them. She was at last baptized in the Spree, and at the time experienced a sort of ecstasy.

We need not trace the history of the case through the details of fanaticism and mysticism patiently collected by Ideler. She saw more spectral illusions; her health began to fail, and at last she was received into the lunatics' hospital, suffering from religious melancholy. After nearly a year's treatment, she wrote her autobiography, and it was so well put together, and her mind and body in other respects were so much improved, that she was dismissed cured.

We have in the preceding article discussed a psychological subject of vast importance, upon which many very mistaken opinions are entertained. In conclusion, we would observe, that an important distinction is to be drawn between these deranged affections of the mind resulting from the influence of false religion upon the understanding, and the healthy effect of legitimate Christianity upon the feelings and actions of man. During the course of our experience, we have never seen a case of insanity which could be clearly traced to true religion—we mean, the religion as inculcated by the great Author of Christianity—the religion that teaches "peace and goodwill towards men"—which advocates the noble sentiments of love and charity—which inculcates the feelings of "preferring others to ourselves"—the religion which represents LOVE, MERCY, and FORGIVENESS as the pre-eminent attributes of the Godhead—the religion whose tendency is to induce us to take lowly views of ourselves, to humble human pride, to produce a cheerful, serene, and happy state of mind—the religion which enables us to bear with fortitude "the whips and scorns of time, the oppressor's wrong, and the proud man's contumely." We cannot believe that the influence of such a religion can be otherwise than salutary in its effects on the human mind.

False, fanatic, and mistaken views of our duty to God and to man, of our relationship to the divine and benevolent Governor of the universe, have decidedly a most pernicious effect upon the feelings and intellect, and often produce unequivocal insanity. How many, with, we believe, the purest intentions, and for the best of motives, represent God, whose great and noblest attribute is LOVE, as a God of vengeance and terror, and who have no conception of the Deity, except as one "riding upon the whirlwind and the storm," hurling his thunderbolts among those transgressing his laws. The inculcation of such doctrines to persons of weak mind, of extreme nervous susceptibilities, or to persons predisposed to insanity, cannot but be otherwise fatal to the integrity of the understanding. How often have we witnessed the lamentable results of such an injudicious course of procedure!

It should, however, never be forgotten, that the mind may seize upon a delusion associated with religion, or it will lay hold of any other fancy, without religion having anything to do with the derangement as an exciting cause. There are thousands of persons capable of mixing in society, and who daily participate in all the enjoyment that a social life can produce, who are hovering on the brink of insanity, and who only require to be exposed to some trifling exciting cause, in order to develop insanity in its worst forms. The "exciting cause" may be an injudicious attempt to proselyte—it may be violent mental emotion, caused by the sudden accession of wealth, or by some unexpected adversity—causes most trifling in their character, often rouse the latent disease or tendencies, and induce positive derangement of mind. Again, when there exists a predisposition to insanity, the mind often seizes hold of some prominent topic of the day, which it exaggerates and falsifies, until it becomes a fixed delusion. The more the matter in question affects the imagination, the more calculated is it to put the mind off its balance. If the person who is unfortunate enough to possess an excitable nervous temperament, or a tendency to derangement of the mind, escape any great mental shock, he may pass through life a healthy, sane man. We have in our "mind's eye" many individuals, who, we conceive, require but some apparently insignificant cause to send them over the precipice. Taking this view of the question, how important is it that the mind should be properly regulated and disciplined; and that correct physiological principles should be promulgated in relation to the prevention of insanity in those constitutionally predisposed to this fearful calamity!

With regard to the prevention of what is termed religious insanity, we should be careful, particularly in early life, when the imagination is most alive to impressions, to avoid allowing the mind to dwell too much on the consideration of the abstractions of religion, and to keep a check upon the feelings. Religious exercise, although all-important, not only in reference to this life, but to a future state of existence, ought to be kept within reasonable and healthy bounds. The mind cannot delusively dwell for any length of time on a subject like that of religion, without running a great risk of disordering its faculties. If the intellect does not become palpably deranged, the mind will be disposed to make false estimates of things, and to attach to trivial and unimportant matters, notions that it is not justified in entertaining.

We cannot unduly exercise any one class of mental operations without weakening the other faculties of the mind—the feelings cannot be exclusively acted upon without enfeebling the powers of reason and judgment; neither can we call these latter faculties, important as they are, into constant operation, leaving the sentiments and propensities in abeyance, without endangering the healthy state of the mind. If the nervous energy, upon which the powers of the mind depend, be concentrated in one part of the brain for too long a period, the other portions of this organ must necessarily suffer, and consequently some of the faculties of the mind become either debilitated, or the ideas distorted. Many of the false views which the world entertains on the important subject of religion owe their origin to this exclusive devotion and consideration of one all-absorbing topic. The feelings and sentiments are educated or exercised at the expense of the judgment, and thus erroneous ideas force themselves upon the mind. He is the happiest and the healthiest man whose mental faculties are equally developed and exercised. Such a person, under the guidance of the Divine Spirit, knows his duty to God and man; and whilst cultivating a cheerful piety, will look with feelings of charity on the failings of his fellow-men, and endeavour, by gentleness and Christian humility, to bring his "erring brother" into the path that leads to virtue, peace, and happiness.

> "True piety is cheerful as the day,
> Will weep indeed, and heave a pitying groan
> For others' woes, but smile upon her own!"

